# Prevalence and location of myofascial trigger points in dogs with osteoarthritis

**DOI:** 10.3389/fvets.2025.1488801

**Published:** 2025-01-15

**Authors:** Maira Rezende Formenton, Denise Tabacchi Fantoni, Lisa Gauthier, Thibaut Cachon, Lin Tchia Yeng, Karine Portier

**Affiliations:** ^1^School of Veterinary Medicine and Animal Science, University of São Paulo, São Paulo, Brazil; ^2^VetAgro Sup (Campus Vétérinaire), Centre de Recherche et de Formation en Algologie Comparée (CREFAC), University of Lyon, Marcy l’Etoile, France; ^3^School of Medicine, Institute of Orthopedics and Traumatology, University of São Paulo, São Paulo, Brazil; ^4^Université Claude Bernard Lyon, Centre de Recherche en Neurosciences de Lyon, INSERM, CRNL U1028 UMR5292, Lyon, France

**Keywords:** myofascial pain, analgesia, muscle pain, myofascial pain syndrome, degenerative joint disease

## Abstract

**Introduction:**

This study was designed to determine the prevalence of myofascial pain and the location of myofascial trigger points (MTPs) in dogs with osteoarthritis.

**Methods:**

Thirty-five dogs were selected and examined for the presence of MTPs using palpation. Assessments were performed independently by two examiners. Correlations between age, MTP number and location, and the site of osteoarthritis were also investigated.

**Results and discussion:**

Thirty out of 35 dogs (86%) had at least one MTP and only 5 (14%) had none. A total of 177 MTPs were identified in dogs in this sample. The prevalence of MTPs was higher in the longissimus thoracicae (40% and 43%; left and right side respectively), followed by the quadriceps femoris (40% and 31%), longissimus lumborum (20% and 23%), gluteus medius and deltoid muscles (14%; left side only), and the pectineus muscle (11%; right side only). The number of osteoarthritic joints was not correlated with the number of MTPs or age. However, age was positively correlated with the number of muscles affected by MTPs. Correlations between the presence of MTPs in muscles surrounding and the affected joints were also lacking.

**Conclusion:**

The prevalence of MTPs in dogs with osteoarthritis is high. Myofascial TPs are positively correlated with age in these patients. The subjective nature of palpation is a major limitation in myofascial pain assessment. Appropriate training and use of standardized diagnostic criteria are recommended.

## 1 Introduction

Myofascial pain syndrome (MPS) is a disorder of muscles, fascia and ligament attachments characterized by (palpable) hypersensitive spots in a taut muscle band. Pain is a cardinal signs of MPS in humans, along with motor and autonomic abnormalities (1, 2). These painful spots are defined as myofascial trigger points (MTPs) and correspond to areas with typical responses to palpation. Classic signs of MPS include localized pain, weakness and a characteristic motor response reflex known as local twitch response (1–3).

Myofascial trigger points may be latent or active. Active points cause spontaneous localized or radiating pain and functional limitations, even when the affected muscle is at rest, whereas latent points do not. Therefore, treatment should be focused on active points (4). Satellite MTPs may develop in the same muscles affected by primary MTPs, in different muscles in the referred pain pathway or in synergistic muscles. Primary MTPs are closely related to motor endplate dysfunction and are typically found around the center of the muscle (5).

The pain on the TP is referred to by human patients as diffuse deep, with a burning sensation and irradiation areas (6). In small animals, vocalizations and attempts to escape are common reactions to the palpation of a TP, associated with avoidance of touch and a twitch reaction (7, 8). In addition, motor dysfunction generates fatigue, muscle weakness, and local sympathetic nervous system alterations (6, 9). The etiology is multifactorial and these areas may appear following muscle damage. This may be the result of direct aggression such as trauma, microaggressions, or chronic inflammation secondary to fatigue (10, 11).

Pain associated with MTPs is described by human patients as diffuse and deep, with a burning sensation and radiation (to other) areas of the body (6). In small animals, vocalization, escape responses, touch sensitivity and (local) twitch response are common reactions to MTP palpation (7, 8). Motor dysfunction also causes fatigue, muscle weakness and local sympathetic nervous system changes (6, 9). Myofascial trigger points may have several causes, such as muscle damage induced by direct trauma, microdamage or chronic inflammation secondary to fatigue (10, 11).

The prevalence of MTPs in small animals has not been determined. However, it seems to be a common, yet underdiagnosed problem, perhaps because vets are often unfamiliar with the condition (12). As a result, treatment tends to be limited to the (primary) orthopedic condition (arthritis, osteoarthritis, bone deformities, herniated discs, etc.); hence the high number of unsuccessful cases. To establish the diagnosis, the examiner must be trained to recognize signs of myofascial pain, palpate tender spots and rule out other conditions (7, 8, 12). Between 21 and 93% of human patients with localized pain are thought to suffer from myofascial pain (13).

Osteoarthritis (OA) can lead to activation of MTPs, leading to increased levels of pain in human patients (14, 15). Despite anatomical and biomechanical differences between human beings and dogs, musculoskeletal conditions such as osteoarthritis and muscle pain share common underlying mechanisms (16, 17) and the same joints (hip, stifle, shoulder and elbow) tend to be affected in both species (18, 19). However, while the prevalence of MTPs in humans with osteoarthritis is well documented (14, 20), there is a limited amount of data in dogs.

One of the few studies investigating MTPs in small animals included 48 lame dogs, 31 of which had not responded to pharmacological treatment. A total of 82 trigger points were identified in these 48 dogs (7). In that sample (7), only eight dogs failed to respond to treatment with dry needling or MTP infiltration. In a different study describing the location of MTPs and methods of palpation for myofascial pain diagnosis in working dogs (8), 72 MTPs were found, primarily in the back and hind limbs.

This study set out determine the prevalence of myofascial pain and the location of MTPs in dogs with osteoarthritis. Correlations between number of MTPs, age and number of affected joints were investigated. The number of MTPs in muscles surrounding affected and non-affected joints was also compared. The following hypotheses were tested: the prevalence of MTPs is high in dogs with osteoarthritis; the number of MTPs increases with age; the number of joints affected with osteoarthritis is correlated with the presence of MTPs in surrounding muscles.

## 2 Methods

### 2.1 Study population

The protocol was approved by the Ethics Committee of VetAgro-Sup (Protocol Number 2115 RECH-ETIC-P003-E01) and the Ethics Committee of the School of Veterinary Medicine and Animal Science, University of São Paulo (Protocol Number 1571120219). Dogs were recruited from the surgery department of VetAgro-Sup (Veterinary Campus of Lyon). Male and female osteoarthritic dogs weighing 15 to 75 kg were included, regardless of the joint or joints affected.

The diagnosis of OA was made by a veterinary orthopedic specialist, based on the following clinical signs and radiologic findings: lameness, joint distention, pain on palpation, decreased range of motion, crepitus, osteophytosis and subchondral sclerosis (21).

Only dogs whose owners have signed a Term of Free and Informed Consent were included. Exclusion criteria were as follows: history or suspicion of neoplasia; dogs aged less than two years; dogs with severe diseases or clinical syndromes (uncontrolled heart disease, liver disease, acute or chronic kidney disease, uncontrolled skin conditions); obese dogs (body condition score higher than eight), as per LaFlamme (22); aggressive dogs; dogs with neurological diseases (peripheral neuropathies, proprioceptive or sensory deficits or seizures) or orthopedic conditions that might interfere with myofascial (pain) assessment (recent surgery, less than 60 days; traumatic hip dislocation, acute trauma, immune mediated or infectious arthritis, dogs submitted to arthroplastic procedures or limb amputation, osteomyelitis, discospondylitis). Dogs receiving steroid or non-steroid anti-inflammatory drugs, analgesics such as tramadol or paracetamol, or recent (less than three months) treatment with gabapentin, pregabalin, amantadine or other adjuvants analgesics were also excluded.

### 2.2 Experimental design

#### 2.2.1 Palpation and identification of MTPs

Selected dogs were palpated by two examiners, one with more than ten years of experience in (canine) physical therapy and one novice trained for one day by the senior examiner in order to standardize the palpation technique.

Criteria for myofascial pain syndrome (diagnosis) and location of active the MTPs were extracted from the human and animal literature (1, 4, 8, 13, 23, 24). The following indicators were used: hyperalgesic points eliciting a typical contractile pain response to palpation, vocalization, rotating the head or gazing towards the palpated area or the examiner, grunting or aggressive reaction to palpation, flexion of the spine to avoid contact or try to escape. We believe these reactions are interpreted as pain responses whenever they are associated with a typical muscle contraction. Myofascial trigger points meeting aforementioned criteria and identified by one or both examiners were recorded on individual evaluation sheets and marked on a reference image showing the corresponding anatomical location. To ensure accurate location of MTPs, large muscle groups such as the latissimus dorsi were subdivided into smaller regions, as shown in Table 1 (8).

**Table 1:** Muscles palpated in different regions of the body in 35 dogs with osteoarthritis.

Region of the body	Muscles palpated
Head and neck	Masseter, brachiocephalicus, sternocephalicus, and trapezius pars cervicalis,
Front limbs	Pectorales superficialis and pectoralis profundus, supraspinatus, infraspinatus, deltoideus, brachioradialis, extensor carpi radialis, extensor digitorum communis, extensor carpi ulnaris, flexor carpi ulnaris, triceps brachii, brachialis, biceps brachii, and omotransversarius.
Back	*Trapezius* (trapezius pars thoracica), latissimus dorsi, longissimus thoracicae, longissimus lumborum, and serratus ventralis thoracis.
Hind limbs	Gluteus superficialis, gluteus medius, biceps femoris, sartorius, tensor fasciae latae, quadriceps femoris, gracilis, semitendinosus, semimembranosus, fibularis peroneus longus and brevis, flexor digitorum superficialis, flexores digitorum profundi, gastrocnemius, extensor digitorum longus, tibialis cranialis, pectineus, and iliopsoas.

The palpation technique employed in this study has been described elsewhere (8). Briefly, it consists of gentle muscle palpation perpendicular to muscle fibers, using the flat or the pinch technique (4, 8).

In areas with different muscle layers (such as the latissimus dorsi and serratus muscles), pressure application was gentle at first, then gradually increased, depending on the muscle layer involved and the activity of the MTP. Palpation was performed using the fingertips or thumb, and contact with the patient’s skin maintained throughout. The underlying muscle was rolled between the tips of the digits until a taut band was detected. That band was then pulled and released, triggering a pain response. All dogs were examined in lateral recumbency.

Examiners were blinded to each other’s findings. Muscle palpation was performed independently, alternating the order of examiners. Examiners were also unaware of the site of osteoarthritis. Head and neck muscles were palpated first and special attention given to the *masseter, brachiocephalicus* and *sternocephalicus*, and the cervical portion of the *trapezius muscle*. Front limbs muscles were palpated next (*pectoralis superficialis* and *profundus, supraspinatus, infraspinatus* and *several others*). Back muscles were then palpated, including the thoracic portion of the *trapezius* and the *latissimus dorsi, longissimus thoracicae, longissimus lumborum* and *serratus ventralis thoracis* muscles. Finally, pelvic limb muscles (*gluteus superficialis, gluteus medius* and others) were palpated.

A total of 41 muscles were palpated (Tables 1, 2). However, when subdivisions of larger muscle groups were accounted for, this number increased to 49 muscle regions. Overall, 98 palpations were performed on 35 dogs (right and left sides), totaling 3,430 assessments.

**Table 2:** Anatomical landmarks used for myofascial trigger point location and subdivision of large muscles.

Muscles	Region	Anatomical reference
Longissimus thoracicae	1	Dorsal to the rib cage, paravertebral, 4th to 9th thoracic vertebra.
	2	Dorsal to the rib cage, paravertebral, 10th to 13th thoracic vertebra.
Longissimus lumborum	1	Dorsal, 1st to 4th lumbar vertebra.
	2	Dorsal, 5th to 7th lumbar vertebra.
Latissimus dorsi	1	4th to 7th rib, laterodorsal aspect, below the trapezius thoracicae muscle.
	2	7th to 10th rib, laterodorsal aspect, below the longissimus dorsi thoraricae muscle.
	3	4th to 7th rib, lateral aspect, below area 1.
	4	7th to 10th rib, lateral aspect, below area 2.
	5	10th to 13th rib, laterodorsal aspect, below the longissimus dorsi thoraricae muscle.
Quadriceps femoris	1	Rectus femoris muscle; vastus lateralis (dorsal portion); vastus medialis (ventral portion).
	2	Vasto intermedius muscle; vastus lateralis (ventral portion); vastus medialis (ventral portion).
Triceps brachii	1	Long head of the triceps brachii muscle (dorsal aspect); lateral head of the triceps brachii muscle; long head of the triceps brachii muscle (ventral aspect).
	2	Long head the triceps brachii muscle (ventral aspect)

Adapted from Formenton et al. (8).

Owners were educated about the primary manifestations of MPS after data collection. Different techniques and methods for treatment and prevention, such as massage, stretching, hot packs and exercise, focusing on the MTPs found on each dog, were explained and demonstrated to enable owners to treat their pets at home.

#### 2.2.2 Statistical analysis

Sample size calculation considered a prevalence of 85% in dogs. The sample size required to achieve a level of confidence of 90% with 10% of error is 35. The prevalence (of MTPs) in each muscle or subdivision was considered an independent measure and used to calculate the following ratio: number affected/total muscle area per side (35 animals). The formula used for sample size estimation was:

$$n=\frac{\rho \left(1-\rho \right)z_{\gamma }^{2}}{\in^{2}}$$

In this formula: n: estimated sample size. *ε*: error. p: expected percentage we wish to find, z_γ_: Normal distribution in the quartile $\frac{1-\gamma }{2}$ . Considering a confidence interval of 90%: z_0.9_ = −1.6448.

Data normality was assessed using the Shapiro–Wilk test. The level of significance was set at 5% (*p* < 0.05).

The percentage of MTPs was calculated per muscle (or region, in the case of large muscle groups). To estimate the prevalence of MTPs in a particular muscle or group of muscles (Table 2), the number of dogs with at least one MTP in that muscle or group of muscles was counted and divided by 35 (number of dogs in the sample, one muscle/group of muscles per dog). This was done for the left and the right side of each dog. For example, if MTPs were found in the right biceps femoris of 2 dogs, the prevalence of MTPs in the right biceps femoris would be 6% (2/35). Calculations were based on data collected by each examiner and their intersection. Each muscle assessment was treated as an independent measure.

Correlations between age and number of MTPs, and age and number of affected joints, were investigated using the Spearman correlation test. The number of MTPs in muscles associated with osteoarthritic and non-affected joints was also compared. Supplementary Table S1 shows which muscles were thought to be associated with each joint.

## 3 Results

**Figure 1:**
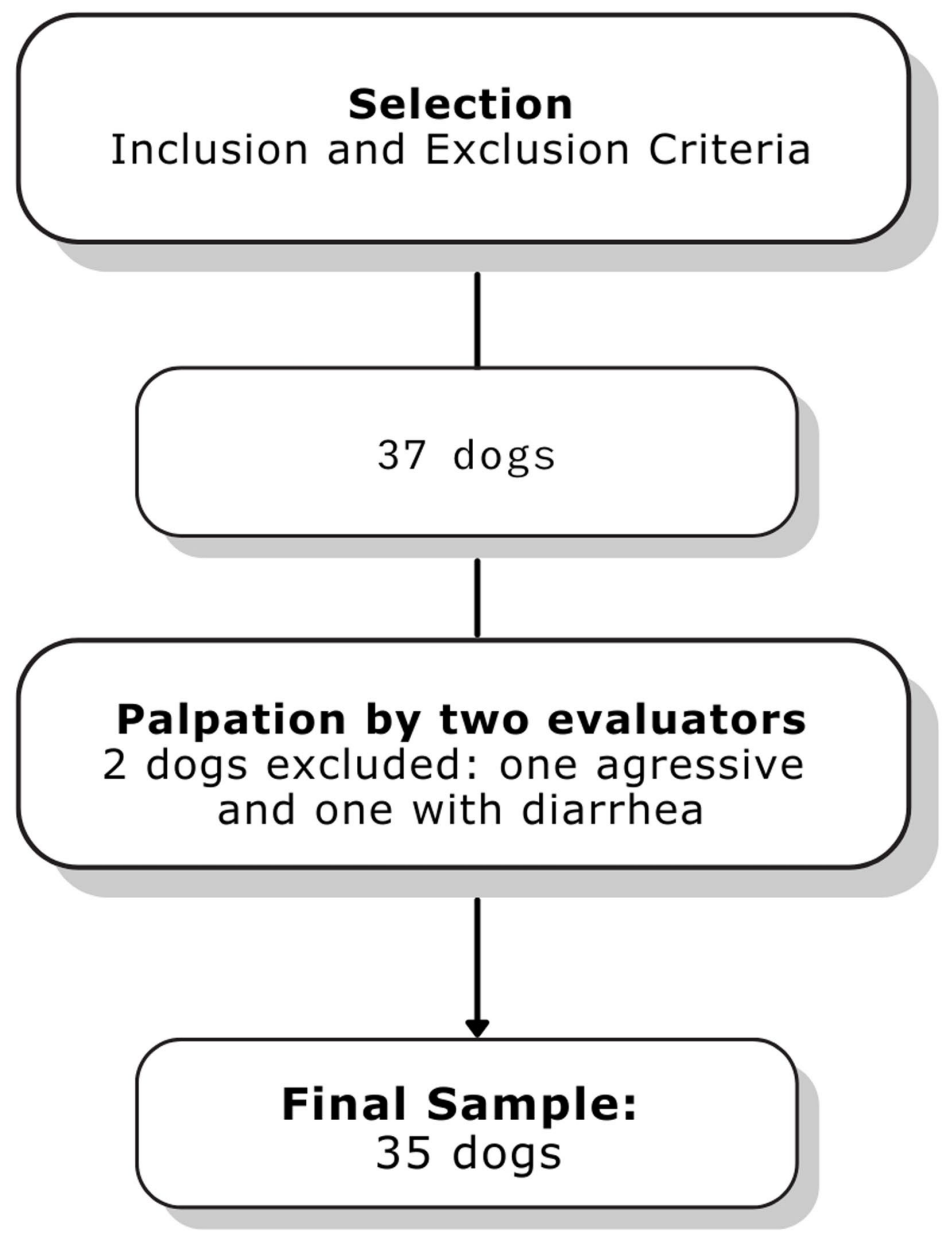
Flowchart of methods and final sample in the study.

Thirty-seven dogs were recruited between April 2021 and July 2021. Two dogs were excluded, one due to diarrhea on the day of examination and one due to aggressive behavior precluding palpation (Figure 1). The final sample comprised 35 dogs, 14 (40%) females and 21 (60%) males aged 6 ± 3.3 years and weighing of 32.2 ± 13 kg on average. The following breeds were represented: mixed-breed (20%), Golden Retriever (14%), Border Collie (11%), Labrador Retriever (6%), Australian Shepherd (6%), German Shepherd (6%), Bernese Mountain Dog (6%) and Brittany Spaniel (6%). Other breeds (Rottweiler, Swiss White Shepherd, French Bulldog, Cane Corso, Auvergne Pointer, Anatolian Shepherd, Staffordshire Bull Terrier, French PointerPointer, and Majorca Mastiff) were represented in small numbers (3% each) and analyzed as a single group (31% overall).

Thirty out of 35 (86%) dogs had at least one MTP, and only 5 (14%) dogs had none. Palpation of 49 muscle regions on each side of 35 dogs revealed a total of 177 MTPs (6 MTPs per dog on average). The number of MTPs per dog is shown in Table 3.

**Table 3:** Number of myofascial trigger points (MTPs), number of affected joints and patient age in a sample of 35 dogs with osteoarthritis.

Dog	MTPs (N)	Joints with OA (N)	Age (years)	Affected joint/joints
1	12	1	10	Left stifle
2	9	1	11	Left stifle
3	4	1	7	Right elbow
4	5	1	3	Right stifle
5	6	3	12	Left stifle, right stifle, left elbow
6	9	2	13	Right elbow, left elbow
7	6	4	4	Tarsal left, right stifle, left hip, right hip
8	8	1	9	Right stifle
9	8	2	9	Right stifle, left elbow
10	3	2	9	Left hip, right hip
11	5	2	8	Left stifle
12	13	1	8	Right stifle
13	0	2	7	Left stifle, right stifle
14	2	2	7	Left stifle, right stifle
15	3	2	7	Right stifle, left stifle
16	5	2	7	Right stifle, left elbow
17	5	2	7	Left hip, right hip
18	5	2	5	Left hip, right hip
19	7	4	5	Left stifle, right stifle, left hip, right hip
20	5	1	6	Right stifle
21	3	3	5	Right stifle, left stifle, left elbow
22	5	2	3	Left hip, right hip
23	10	3	7	Left stifle, left hip, right hip
24	0	2	7	Left hip, right hip
25	9	3	14	Left shoulder, right stifle, left stifle
26	4	3	4	Left hip, right hip, left stifle
27	0	2	4	Right elbow, left elbow
28	6	1	4	Right elbow
29	5	2	3	Left hip, right hip
30	2	1	2	Right stifle
31	0	1	3	Left elbow
32	1	2	3	Left elbow, right elbow
33	5	2	7	Left stifle
34	0	2	2	Left hip, right hip
35	7	5	14	Left elbow, left hip, right hip, right stifle, left stifle

**Figure 2:**
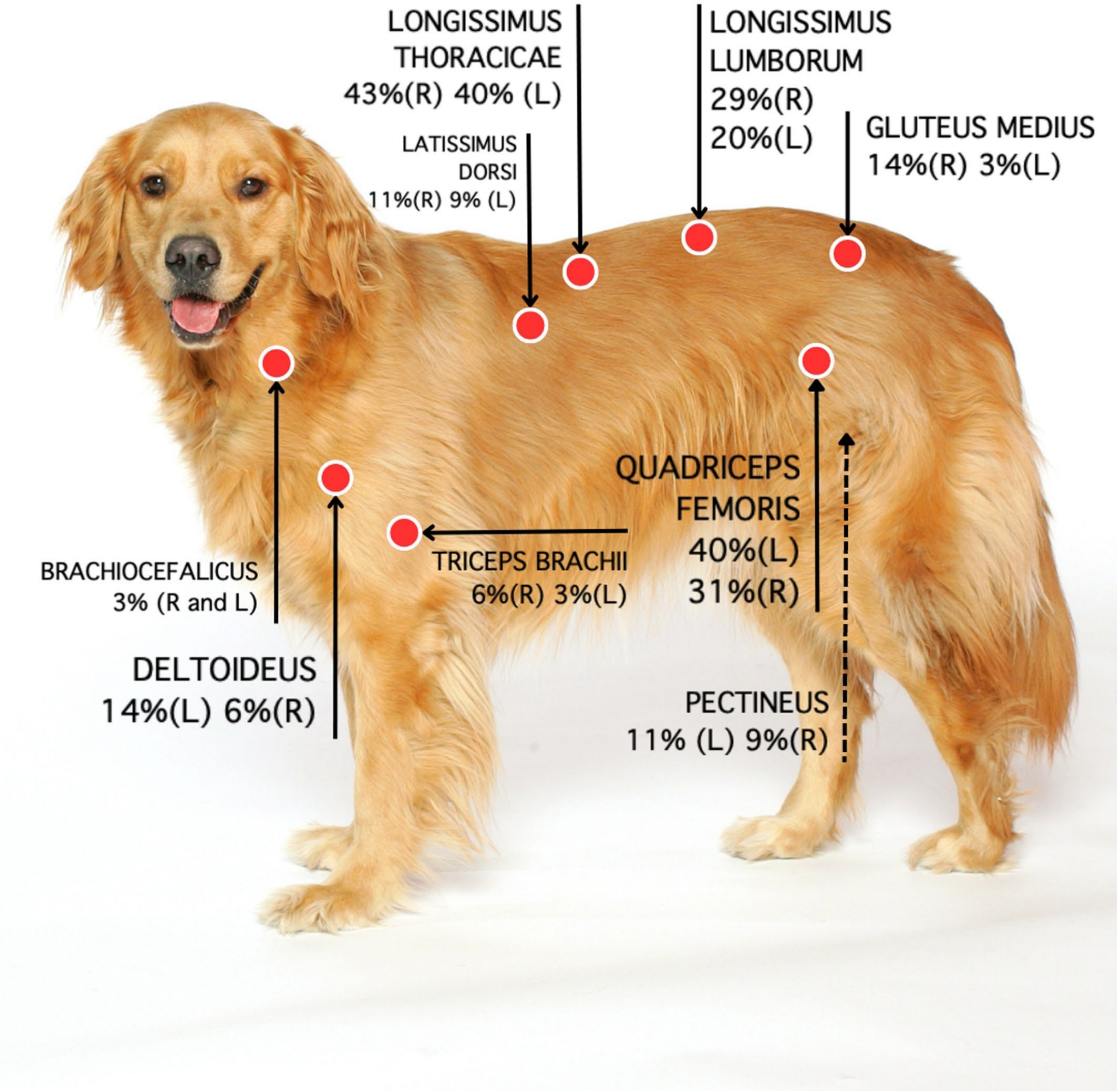
Location and percentage of MTPs based on data collected by two examiners. Each muscle assessment was treated as an independent measure. R: Right side. L: Left side.

Ninety (51%) out of 177 MTPs were located on the left (L) and 87 (49%) on the right (R) side. Most MTPs (95/177; 54%) were located on the back. Remaining MTPs were located in hind limb muscles (64; 36%), front limb muscles (16; 9%) or head and neck muscles (2; 1%) (Table 4). The longissimus thoracicae muscle (region 1) had the highest prevalence of MTPs, followed by the quadriceps femoris (region 2), longissimus thoracicae (region 2), longissimus lumborum (region 2), gluteus medius, deltoid and pectineus muscles. The prevalence of MTPs in major muscle regions on each side of the body is shown in Figure 2. The location and prevalence of all MTPs are described in Table 4. An anatomical version of Figure 2 is shown in Supplementary material.

**Table 4:** Number and prevalence of myofascial trigger points (MTPs) in different muscle groups examined in 35 dogs with osteoarthritis.

Muscle group or region	Number and prevalence of MTPs			
	Left		Right	
Biceps femoralis	2	6%	2	6%
Brachiocephalicus	1	3%	1	3%
Deltoideus	5	14%	2	6%
Extensor carpi radialis	1	3%		
Gastrocnemius			1	3%
Gluteus medius	5	14%	1	3%
Gracilis			1	3%
Iliopsoas			1	3%
Latissimus dorsi 1	3	9%	4	11%
Latissimus dorsi 2	2	6%		
Latissimus dorsi 3	2	6%	3	9%
Latissimus dorsi 4	1	3%	1	3%
Longissimus lumborum part 1	10	29%	7	20%
Longissimus lumborum part 2	7	20%	8	23%
Longissimus thoracicae part 1	14	40%	15	43%
Longissimus thoracicae part 2	9	26%	9	26%
Pectineus	4	11%	3	9%
Pectoralis superficialis			1	3%
Quadriceps femoris 1	2	6%	8	23%
Quadriceps femoris 2	14	40%	11	31%
Sartorius	3	9%	3	9%
Semitendinosus			2	6%
Tibialis cranialis			1	3%
Trapezius pars thoracica	4	11%		
Triceps brachii 2	1	3%	2	6%
MTPs per side*	90	51%	87	49%

Independent muscle assessments performed by 2 examiners were treated as an independent measure. Frequency was expressed as number of muscles affected/number of muscle groups or regions examined per side (35 dogs). *Percentage differences between the left and right sides are shown at the bottom of the table.

**Figure 3:**
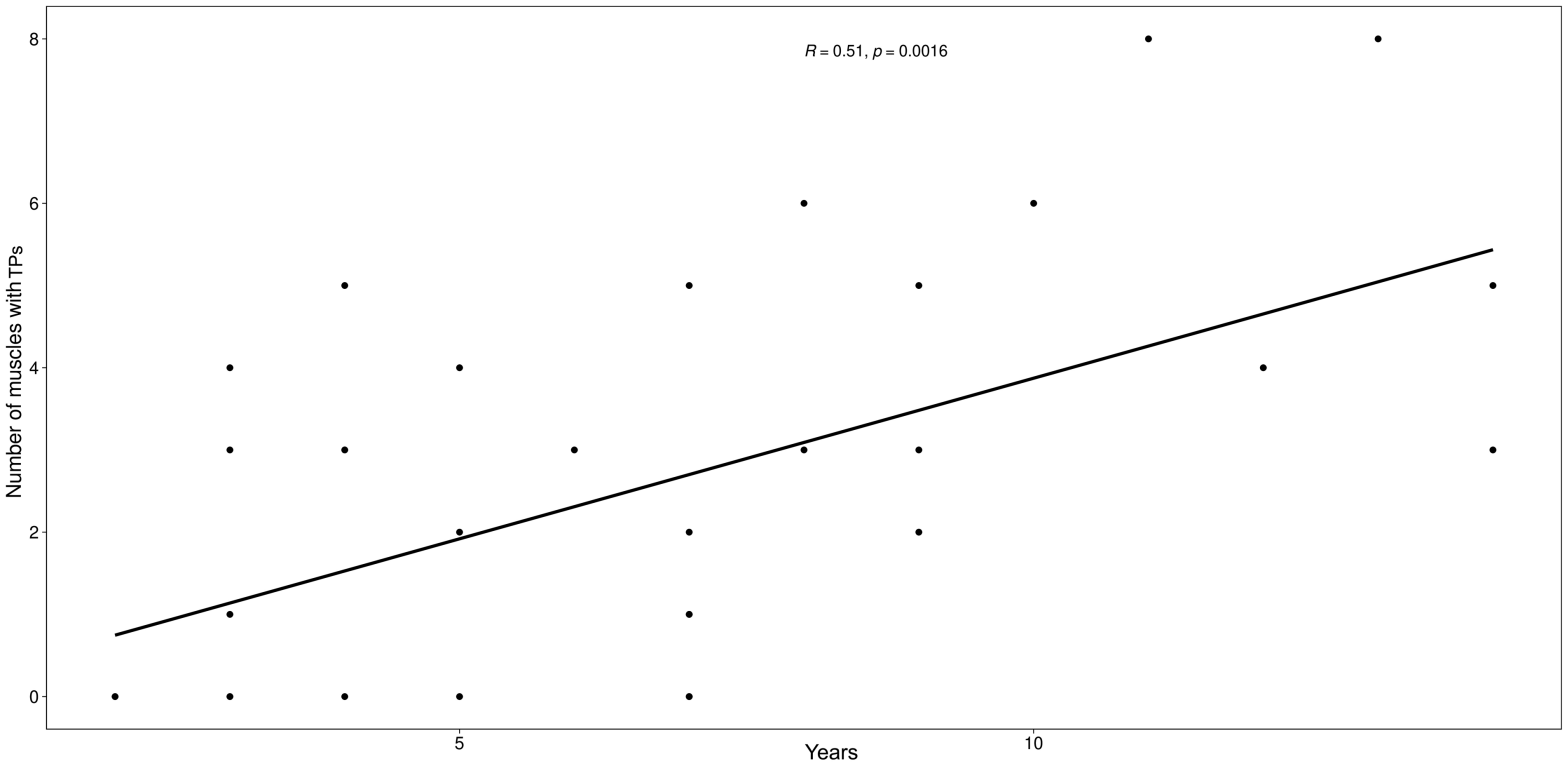
Scatter plot between the number of muscles with MTPs and the age (years) of the animals. The graph illustrates the positive relationship between the two variables, establishing that the number of muscles affected by trigger points increases as age increases.

Age was positively correlated with the number of MTPs per dog (*r*^2^ = 0.56, *p* = 0.0005; Figure 3) but not with the number of osteoarthritic joints (*r*^2^ = 0.23, *p* = 0.1772). The number of MTPs was not correlated with the number of osteoarthritic joints per dog (*r*^2^ = 0.04, *p* = 0.8021).

The number of MTPs in muscles surrounding affected and non-affected joints was also compared. Comparative data are shown in Table 5 and Supplementary Table S3. The percentage of dogs with OA and MTPS is 1.81%. Myofascial TP location was not correlated with the presence of OA (Table 5).

**Table 5:** Presence of osteoarthritis (OA) and presence of myofascial trigger points (MTPs) in related muscles detected by at least one examiner (*p* < 0.001).

OA	Absence of MTPs	Presence of MTPs	Number of assessments
No	3,253 (94.87%)	29 (0.85%)	3,282 (95.71%)
Yes	85 (2.48%)	63 (1.81%)	148 (4.29%)
Total	3,338 (97.35%)	92 (2.65%)	3,430 (100%)

## 4 Discussion

This is the first study to investigate the prevalence of MTPs in dogs with osteoarthritis. As in humans (14), the prevalence is high (86%) and the number of MTPs seems to increase with age.

Sánchez-Romero et al. (14) reported a high prevalence of MTPs in humans with OA, up to 50% in the tensor fasciae latae. Higher prevalence of MTPs has also been observed in working dogs (8). Some theories may explain the mechanisms underlying pain and disability in patients with *OA* and myofascial pain. Trigger points cause central sensitization, increasing joint pain perception in humans (25–27). Higher concentrations of allogenic substances and pro-inflammatory mediators in patients with active MTPs, both locally and in non-affected or distant muscles (28, 29), suggest a systemic inflammatory condition.

Increased nociceptive transmission in spinal cord segments associated with areas with active compared to latent MTPs has also been reported (30), showing that pain arising from active MTPs may impact the central perception of pain, enhancing pain perception overall, including osteoarthritis-related pain (15, 27).

Central sensitization reflects the ability of MTPs to induce changes in the posterior horn of the dorsal spinal cord (PHDSC) due to the high plasticity and connectivity of the nervous system in this area (31). Changes in the PHDSC enhance the expression of glutamate receptors mGluR1α/mGluR5/NMDAR1 in regions associated with MTPs. A-fibers are thought to be involved in the development and maintenance of central sensitization (32). At the central nervous system level, abnormal activity has been demonstrated in the amygdala, central cingulate gyrus, lower parietal cortex and middle portion of the insula, suggesting that pain arising from MTPs is processed in different parts of the brain (33). Hence the increased sensitivity to pain in veterinary and human patients (33–35).

The high prevalence of MPS in dogs with osteoarthritis may also be explained by excessive mechanical stimulation at the muscular level (36) and muscle overload resulting from biomechanical and pathophysiological changes induced by osteoarthritis (37). Vesicles in free nerve endings in muscle nociceptors contain substance P and calcitonin gene related peptide (CGRP). When activated by mechanical or chemical stimuli, these substances are released and nerve conduction occurs (35). Substance P and CGRP induce microvascular vasodilation, triggering the release of pro-inflammatory substances such as histamine, serotonin, prostaglandins and bradykinin (38), all of which induce muscular injury (39) and fascial contraction (40), leading to the formation of painful spots (41).

Also, reflex microvascular vasoconstriction and reduced blood flow at the site of MTPs in response to CGRP and substance P-induced vasodilation reduce tissue oxygenation, further complicating the metabolic crisis (35). These mediators not only trigger the inflammatory process but also damage local muscle fibers, again stimulating free nerve endings in muscle nociceptors and perpetuating the neuromuscular inflammation cycle (11, 35, 38). Tissue damage also stimulates tumor necrosis factor alpha (TNF-*α*) and interleukin production, increasing peripheral sensitization (42). High concentrations of substance P, bradykinin, CGRP, TNF-α, interleukins and norepinephrine at the site of active MTPs have been demonstrated and support the proposed cyclic mechanism of muscle injury (28, 34).

The impact of MTPs in patients with OA has been widely reported (20, 43) and treatment of the myofascial component improves pain experience in these patients (14, 44). Indeed, chronic pain is one of the most common symptoms and a major contributing factor to poor quality of life in OA patients (25). Soft tissue-related pain may also cause discomfort and disability; therefore, the affected joint is not the only source of pain in human patients with OA (15, 45–47).

In this study, most MTPs were found in back and hind limb muscles, the longissimus and quadriceps femoris being the most commonly affected. Therefore, these muscles must be examined for MTPs in patients osteoarthritis pain. The most common MTP location in our sample did not differ from the location reported in working dogs (8). In working dogs, the lumbar portion of the longissimus dorsi had the highest prevalence (42%) of MTPs, followed by the latissimus dorsi, the pectineus, the quadriceps femoris and the sartorius muscles (33% respectively) (8).

In a study conducted by Janssens et al. (7), 48 lame dogs were examined and 82 MTPs detected by palpation (2 per dog on average). The average number of MTPs in this sample was 6 per dog. The cause of lameness was not reported in that study (7); hence (direct) comparisons cannot be made. However, as in osteoarthritic dogs in this sample, MTPs were found in the quadriceps femoris and paravertebral muscles. These muscles are possibly the first to be affected by compensatory overload in dogs with orthopedic diseases or submitted to intense physical activities and should be the (primary) focus orthopedic or pain assessment in these animals.

Even in human patients who can describe pain verbally, distinguishing between latent and active or satellite and primary MTPs is challenging (48, 49). Myofascial trigger points identified in this study were probably active and a cause of pain in affected animals, since pain reaction was one of the diagnostic criteria employed. Still, further studies are warranted to establish criteria for MTP diagnosis in animals.

Of note, dogs with OA and myofascial syndrome may be more prone to painful conditions such as chronic pain in the caudal lumbar musculature. In fact, MPS is the leading cause of low back pain in humans (50, 51) and has been associated with pain in OA patients (14, 15, 20).

Interestingly, age was positively correlated with the number of MTPs in this study. However, it cannot be argued that older dogs experienced more pain. In human medicine there is also a lack of consensus regarding increased levels of pain in elderly patients, even though pain is thought to be more common in the elderly population due to the higher prevalence of degenerative diseases such as OA, diabetes and cancer (52).

An epidemiological study investigating pain in the elderly population revealed a higher prevalence of somatic musculoskeletal pain in the early stages of aging and a paradoxical decrease in painful conditions above the age of 80 (53). The reported prevalence of chronic joint pain was 43.0, 60.6, 45.2, and 25.2% in individuals aged 70, 78, 85 and 90 years, respectively. However, in individuals aged over 90, cognitive impairment may affect the ability to self report pain (54). In this study, the number of MTPs increased with age. Further studies are needed to determine whether the number of MTPs is correlated with pain intensity in dogs with OA.

Contrary to our hypothesis and different from findings in humans (55, 56), the number of joints with OA was not correlated with age in this sample, although the sample may not have been adequately powered to do so. The relationship between OA and age is unclear in dogs. According to recent studies, osteoarthritis can be diagnosed at any age (19) and other factors, such as conformation, joint dysplasia and congenital orthopedic conditions are more significant risk factors than age in the canine population (18). The fact that OA is often diagnosed when the disease advanced and pain intensity is high may explain why the disease is not commonly reported in young dogs (57).

The frequency of MTPs in muscles surrounding osteoarthritic and non-affected joints was similar. This finding is not congruent with the human literature (20, 43, 56, 58). However, in this study correlations were established based on anatomic proximity, for lack of a better method. The understanding of quadrupedal gait biomechanics has advanced a lot in recent years (59); muscle activation in the canine stride cycle has also been recently investigated (59, 60). Muscle activation in response to joint motion involves structures other than surrounding muscles, and different activities require whole-body involvement (61).

Osteoarthritis has been shown to affect muscle activation in humans (62). Similar findings have been reported in a superficial electromyography study done in dogs with hip osteoarthritis (63). However, MTPs may be associated with a variety of muscle injuries and do not reflect exclusively changes in muscle activation, as previously discussed. Myofascial kinetic chains have recently been mapped in dogs (40, 64, 65) and may partially explain our results.

Myofascial trigger points may also reflect postural changes and compensatory muscle overload in dogs. The fact that OA may interfere with weight bearing, leading to biomechanical and compensatory changes (66, 67), may explain the apparently random distribution of MTPs in dogs in this sample. As a consequence, a comprehensive whole-body muscle assessment is required in dogs with OA-related pain, regardless of the joint or joints affected. High precision methods such as electromyography can be used for in-depth investigation of the impacts of myofascial pain in affected muscles. Dogs in this sample did not have acute joint pain (OA flare-up). This may have impacted the number of MTPs identified, as dogs with painful joints are likely to present with more MTPs. Future studies with dogs with osteoarthritis and joint pain are recommended to investigate this relationship.

Standardized assessment procedures used in this study have been described elsewhere (8). However, our findings emphasize the subjective nature of palpation, a major limitation which introduces a significant challenge in myofascial pain research reproducibility. Similar difficulties have been described in human studies (49). As recommended in the human literature (24), MTPs were diagnosed by two trained examiners using palpation. However, sequential palpation is also thought to introduce a bias, since the first palpation may interfere with the next, even when the order of examiners is randomized.

Inclusion of dogs with OA in different or multiple joints is yet another limitation of this study. As in some humans (14, 68), investigations with a more specific patient population (a single joint affected or a single cause, such as osteoarthritis secondary to hip dysplasia) is warranted for a more accurate mapping of MTPs in dogs.

## 5 Conclusion

The prevalence of MTPs in dogs with osteoarthritis seems to be high. Most dogs (86%) had at least one MTP, the longissimus and quadriceps femoris being the most commonly affected muscles. The number of MTPs increases with age. However, the number of osteoarthritic joints is not correlated with age or the number of MTPs. There were no correlations of MTPs in muscles surrounding the osteoarthritic joints, probably due to quadrupedal biomechanics. Hence the need of a comprehensive assessment of OA patients, regardless of the joint or joints affected. Findings of this study emphasize the significance of myofascial pain in dogs with osteoarthritis and suggest that MTP treatment may improve patient wellbeing and comfort. Further studies are warranted to explore the potential therapeutic implications of and devise strategies for myofascial pain management in dogs with OA.

## Data Availability

The original contributions presented in the study are included in the article/Supplementary material, further inquiries can be directed to the corresponding author.
