# Supplementary material for: Prevalence and location of myofascial trigger points in dogs with osteoarthritis
Source: Front Vet Sci. 2025 Jan 15;12:1488801. doi: 10.3389/fvets.2025.1488801 (PMC11776090; doi:10.3389/fvets.2025.1488801)
Supplement: Supplementary file 1 [file Data_Sheet_1.PDF]

Supplementary Material

1 Supplementary Figure 1: Main TPs with an anatomical reference of its location.

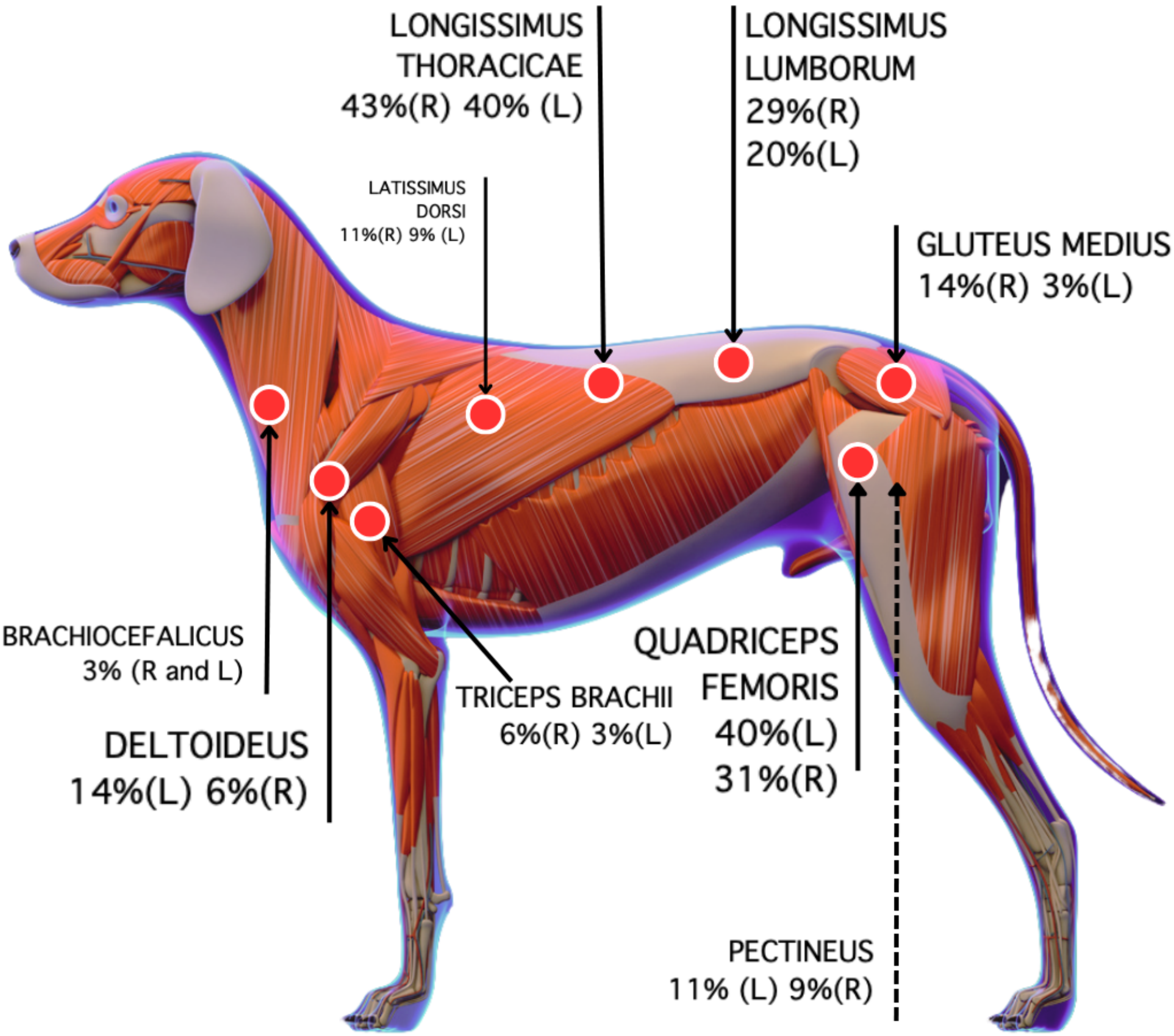

2 Supplementary Tables

2.1 **Supplementary Table 1:** Joint and muscle considered related to it. TPs: Trigger Points.

**Joint**	**Muscles considered associated to this joint for the research of TPs.**
**LEFT SHOULDER**	Masseter<br>Brachiocephalicus<br>Sternocephalicus<br>Trapezius pars cervicalis<br>Pectorales superficialis<br>Pectoralis profundus<br>Supraspinatus<br>Infraspinatus<br>Trapezius pars thoracica<br>Latissimus dorsi<br>Longissimus thoracicae<br>Serratus ventralis thoracis.
**LEFT ELBOW**	Deltoideus<br>Brachioradialis<br>Triceps brachii<br>Brachialis<br>Biceps brachii<br>Omotransversarius
**LEFT CARPUS**	Extensor carpi radialis<br>Extensor digitorum communis<br>Extensor carpi ulnaris<br>Flexor carpi ulnaris
**RIGHT SHOULDER**	Masseter<br>Brachiocephalicus<br>Sternocephalicus<br>Trapezius pars cervicalis<br>Pectorales superficialis<br>Pectoralis profundus<br>Supraspinatus<br>Infraspinatus<br>Trapezius pars thoracica<br>Latissimus dorsi<br>Longissimus thoracicae<br>Serratus ventralis thoracis.
**RIGHT ELBOW**	Deltoideus

	Brachioradialis<br>Triceps brachii<br>Brachialis<br>Biceps brachii<br>Omotransversarius
**RIGHT CARPUS**	Extensor carpi radialis<br>Extensor digitorum communis<br>Extensor carpi ulnaris<br>Flexor carpi ulnaris
**LEFT HIP**	Longissimus lumborum<br>Gluteus superficialis<br>Gluteus medius<br>Pectineus<br>Iliopsoas
**LEFT STIFLE**	Biceps femoris<br>Sartorius<br>Tensor fasciae latae<br>Quadriceps femoris<br>Gracilis<br>Semitendinosus<br>Semimembranosus
**LEFT STIFLE**	Peroneus longus<br>Peroneus brevis<br>Flexor digitorum superficialis<br>Flexores digitorum profundi<br>Gastrocnemius<br>Extensor digitorum longus<br>Tibialis cranialis<br>Fibularis
**RIGHT HIP**	Longissimus lumborum<br>Gluteus superficialis<br>Gluteus medius<br>Pectineus<br>Iliopsoas
**RIGHT STIFLE**	Biceps femoris<br>Sartorius<br>Tensor fasciae latae<br>Quadriceps femoris<br>Gracilis<br>Semitendinosus<br>Semimembranosus
**RIGHT TARSUS**	Peroneus longus<br>Peroneus brevis<br>Flexor digitorum superficialis

Flexores digitorum profundi
Gastrocnemius
Extensor digitorum longus
Tibialis cranialis
Fibularis

2.2 Supplementary Table 2: Location of trigger points on each dog, considering each evaluator and side. TP: Trigger Point.

Dog	Muscle	Side	Evaluator 1	Evaluator 2
1	LONGISSIMUS LUMBORUM PART 1	RIGHT	x	
	QUADRICEPS FEMORIS 1	RIGHT	x	
	QUADRICEPS FEMORIS 2	RIGHT	x	x
	ILIOPSOAS	RIGHT	x	x
	LONGISSIMUS LUMBORUM PART 1	LEFT	x	
	DELTOIDEUS	LEFT	x	x
	QUADRICEPS FEMORIS 1	LEFT	x	x
	PECTINEUS	LEFT		x
2	LATISSIMUS DORSI 1	RIGHT	x	x
	QUADRICEPS FEMORIS 2	RIGHT	x	x
	PECTINEUS	RIGHT		x
	LATISSIMUS DORSI 1	LEFT	x	x
	QUADRICEPS FEMORIS 2	LEFT	x	x
	BICEPS FEMORIS	LEFT	x	
3	LATISSIMUS DORSI 1	RIGHT	x	
	QUADRICEPS FEMORIS 2	RIGHT	x	
	SEMITENDINOSUS	RIGHT		x
	LATISSIMUS DORSI 4	LEFT	x	
	GLUTEUS MEDIUS	LEFT	x	
4	No TP			
5	LONGISSIMUS THORACICAE PART 2	RIGHT	x	x
	LONGISSIMUS LUMBORUM PART 2	RIGHT	x	x
	LONGISSIMUS THORACICAE PART 2	LEFT	x	x
	LONGISSIMUS LUMBORUM PART 2	LEFT	x	x
	QUADRICEPS FEMORIS 1	LEFT	x	
6	LATISSIMUS DORSI 3	RIGHT	x	
	LONGISSIMUS THORACICAE PART 2	RIGHT	x	x
	LONGISSIMUS THORACICAE PART 1	RIGHT		x
	LONGISSIMUS LUMBORUM PART 2	RIGHT	x	
	GLUTEUS MEDIUS	RIGHT	x	x
	QUADRICEPS FEMORIS 2	RIGHT	x	
	EXTENSOR CARPI RADIALIS	LEFT	x	

	**LATISSIMUS DORSI 2**	**LEFT**	x	
	**LONGISSIMUS THORACICAE PART 2**	**LEFT**	x	x
	**SARTORIUS**	**LEFT**	x	
	**QUADRICEPS FEMORIS 2**	**LEFT**	x	
	**GLUTEUS MEDIUS**	**LEFT**	x	x
	**LONGISSIMUS LUMBARUM PART 2**	**LEFT**		x
**7**	**No TP**			
	**DELTOIDEUS**	**RIGHT**	x	
	**LONGISSIMUS THORACICAE PART 2**	**RIGHT**	x	
**8**	**PECTINEUS**	**RIGHT**	x	
	**DELTOIDEUS**	**LEFT**	x	
	**LONGISSIMUS THORACICAE PART 2**	**LEFT**	x	
	**QUADRICEPS FEMORIS 2**	**RIGHT**	x	x
**9**	**QUADRICEPS FEMORIS 2**	**LEFT**	x	x
	**PECTINEUS**	**LEFT**	x	
	**LONGISSIMUS THORACICAE PART 1**	**RIGHT**	x	x
	**LONGISSIMUS LUMBORUM PART 1**	**RIGHT**	x	
**10**	**QUADRICEPS FEMORIS 2**	**RIGHT**	x	x
	**LONGISSIMUS THORACICAE PART 1**	**LEFT**	x	x
	**BICEPS FEMORIS**	**LEFT**	x	
	**PECTORALIS SUPERFICIALIS**	**RIGHT**		x
	**BICEPS FEMORIS**	**RIGHT**	x	x
	**QUADRICEPS FEMORIS 1**	**RIGHT**	x	
**11**	**DELTOIDEUS**	**RIGHT**	x	
	**GLUTEUS MEDIUS**	**LEFT**	x	x
	**DELTOIDEUS**	**LEFT**		x
	**QUADRICEPS FEMORIS 2**	**LEFT**	x	
**12**	**No TP**			
	**QUADRICEPS FEMORIS 1**	**RIGHT**	x	
**13**	**LONGISSIMUS LUMBORUM PART 1**	**RIGHT**	x	x
	**QUADRICEPS FEMORIS 2**	**LEFT**	x	
	**LONGISSIMUS LUMBORUM PART 2**	**RIGHT**	x	x
	**SARTORIUS**	**RIGHT**	x	
**14**	**LONGISSIMUS THORACICAE PART 2**	**LEFT**	x	
	**LONGISSIMUS LUMBORUM PART 2**	**LEFT**	x	
	**SARTORIUS**	**LEFT**	x	x
**15**	**LONGISSIMUS LUMBORUM PART 2**	**RIGHT**	x	x
	**QUADRICEPS FEMORIS 2**	**RIGHT**	x	x
**16**	**NO TP**			
	**LONGISSIMUS THORACICAE PART 1**	**RIGHT**	x	x
**17**	**LONGISSIMUS THORACICAE PART 1**	**LEFT**	x	x
	**QUADRICEPS FEMORIS 2**	**LEFT**	x	
	**LONGISSIMUS LUMBORUM PART 1**	**LEFT**		x

18	LATISSIMUS DORSI 3	RIGHT		x
	QUADRICEPS FEMORIS 2	RIGHT	x	
	LONGISSIMUS THORACICAE PART 1	RIGHT	x	x
	SARTORIUS	RIGHT		x
	PECTINEUS	RIGHT	x	
	BICEPS FEMORALIS	RIGHT		x
	GASTROCNEMIUS	RIGHT	x	
	LONGISSIMUS THORACICAE PART 1	LEFT		x
	QUADRICEPS FEMORIS 2	LEFT	x	x
	LATISSIMUS DORSI 3	LEFT	x	
	LONGISSIMUS LUMBORUM PART 1	LEFT	x	
	PECTINEUS	LEFT	x	
19	LONGISSIMUS THORACICAE PART 1	RIGHT	x	
	LONGISSIMUS THORACICAE PART 1	LEFT	x	
20	LONGISSIMUS THORACICAE PART 1	RIGHT	x	
	LONGISSIMUS LUMBORUM PART 1	RIGHT	x	x
	QUADRICEPS FEMORIS	RIGHT	x	
	LONGISSIMUS THORACICAE PART 1	LEFT	x	x
	LONGISSIMUS LUMBORUM PART 1	LEFT	x	
21	LONGISSIMUS THORACICAE PART 1	RIGHT	x	x
	SARTORIUS	RIGHT		x
	SARTORIUS	LEFT	x	x
	LONGISSIMUS THORACICAE PART 1	LEFT	x	
	LONGISSIMUS LUMBORUM PART 1	LEFT		x
22	LONGISSIMUS THORACICAE PART 2	RIGHT	x	x
	QUADRICEPS FEMORIS 1	RIGHT	x	
	GRACILIS	RIGHT	x	
	TRAPEZIUS THORACICAE	LEFT	x	
	BRACHIOCEPHALICUS	LEFT	x	
	LONGISSIMUS THORACICAE PART 2	LEFT	x	x
	PECTINEUS	LEFT	x	
	QUADRICEPS FEMORIS 2	LEFT		x
23	QUADRICEPS FEMORIS 2	RIGHT	x	
	LONGISSIMUS THORACICAE PART 1	RIGHT	x	
	QUADRICEPS FEMORIS 2	LEFT	x	
	LONGISSIMUS THORACICAE PART 1	LEFT	x	
24	LATISSIMUS DORSI 3	RIGHT	x	
	LONGISSIMUS THORACICAE PART 1	RIGHT	x	x
	LONGISSIMUS LUMBORUM PART 1	RIGHT		x
	LATISSIMUS DORSI 1	LEFT	x	
	LONGISSIMUS THORACICAE PART 1	LEFT	x	x
	LONGISSIMUS LUMBORUM PART 1	LEFT		x

25	LONGISSIMUS LUMBORUM PART 1	LEFT	x	x
26	LONGISSIMUS THORACICAE PART 2	RIGHT	x	x
	TIBIAL CRANIALIS	RIGHT	x	
	LONGISSIMUS THORACICAE PART 2	LEFT	x	x
	DELTOIDEUS	LEFT	x	
	QUADRICEPS FEMORIS 2	LEFT		x
27	No TP			
28	LONGISSIMUS THORACICAE PART 1	RIGHT	x	x
	LATISSIMUS DORSI 1	RIGHT	x	
	TRICEPS BRACHII 1	RIGHT	x	
	QUADRICEPS FEMORIS 1	RIGHT	x	
	LONGISSIMUS THORACICAE PART 1	LEFT	x	x
	LATISSIMUS DORSI 3	LEFT	x	x
	TRICEPS BRACHII 2	LEFT	x	
29	LONGISSIMUS THORACICAE PART 2	RIGHT	x	
	LONGISSIMUS THORACICAE PART 2	LEFT	x	
	QUADRICEPS FEMORIS 2	LEFT	x	
30	LONGISSIMUS THORACICAE PART 1	RIGHT	x	
	LONGISSIMUS LUMBORUM PART 1	RIGHT	x	x
	SEMITENDINOSUS	RIGHT	x	x
	LONGISSIMUS THORACICAE PART 1	LEFT	x	
	LONGISSIMUS LUMBORUM PART 1	LEFT	x	x
	QUADRICEPS FEMORIS 2	LEFT	x	
31	TRICEPS BRACHII 1	RIGHT	x	
	LONGISSIMUS THORACICAE PART 1	RIGHT	x	x
	LATISSIMUS DORSI 1	RIGHT		x
	LONGISSIMUS LUMBORUM PART 2	RIGHT		x
	QUADRICEPS FEMORIS 1	RIGHT		x
	QUADRICEPS FEMORIS 2	LEFT	x	x
	LONGISSIMUS THORACICAE PART 1	LEFT	x	x
	TRAPEZIUS THORACICAE	LEFT		x
	LATISSIMUS DORSI 1	LEFT	x	x
32	LONGISSIMUS THORACICAE PART 2	RIGHT	x	
	QUADRICEPS FEMORIS 1	RIGHT	x	
	LONGISSIMUS THORACICAE PART 2	LEFT	x	
	GLUTEUS MEDIUS	LEFT	x	
	LONGISSIMUS LUMBORUM PART 2	LEFT		x
33	BRACHIOCEPHALICUS	RIGHT	x	
	LONGISSIMUS THORACICAE PART 1	RIGHT	x	
	LONGISSIMUS THORACICAE PART 2	RIGHT		x
	QUADRICEPS FEMORIS 2	RIGHT		x
	LONGISSIMUS LUMBORUM PART 2	RIGHT	x	x
	DELTOIDEUS	LEFT	x	

	TRAPEZIUS THORACICAE	LEFT	x	
	LONGISSIMUS THORACICAE PART 1	LEFT	x	x
	LONGISSIMUS LUMBARUM PART 2	LEFT	x	x
34	LONGISSIMUS THORACICAE PART 1	RIGHT	x	x
	LONGISSIMUS LUMBORUM PART 1	RIGHT	x	x
	LONGISSIMUS LUMBORUM PART 2	RIGHT	x	x
	LATISSIMUS DORSI 4	RIGHT	x	x
	TRAPEZIUS THORACICAE	LEFT		x
	LONGISSIMUS THORACICAE PART 1	LEFT	x	
	LONGISSIMUS LUMBORUM PART 1	LEFT		x
	LONGISSIMUS LUMBORUM PART 2	LEFT	x	x
	LATISSIMUS DORSI 2	LEFT	x	x
35	QUADRICEPS FEMORIS 2	RIGHT	x	
	LONGISSIMUS THORACICAE PART 1	RIGHT	x	x
	LONGISSIMUS THORACICAE PART 2	RIGHT		x
	LONGISSIMUS LUMBORUM PART 2	RIGHT	x	x
	LONGISSIMUS LUMBORUM PART 1	LEFT	x	x
	LONGISSIMUS LUMBORUM PART 2	LEFT		x
	LONGISSIMUS THORACICAE PART 1	LEFT	x	
	LONGISSIMUS THORACICAE PART 2	LEFT	x	
	GLUTEUS MEDIUS	LEFT	x	
	QUADRICEPS FEMORIS 2	LEFT	x	

2.3 Supplementary Table 3: Relation between affected joints and the number of TPs (177 in total) in the muscles related by proximity. TP: Trigger Points. OA: Osteoarthritis.

Joint	Number affected joints in the 35 dogs	Muscles considered associated to this joint for the research of TPs	Joint Affected by OA	Joint Free of OA
			Number of tps in these muscles	Number of tps in these muscles
LEFT SHOULDER	1	MASSETER	**0**	**0**
		BRACHIOCEPHALICUS	**1**	**0**
		STERNOCEPHALICUS	**0**	**0**
		TRAPEZIUS PARS CERVICALIS	**0**	**0**
		PECTORALES SUPERFICIALIS	0	0
		PECTORALIS PROFUNDUS	0	0
		SUPRASPINATUS	0	0

		INFRASPINATUS	0	0
		TRAPEZIUS PARS THORACICA	1	3
		LATISSIMUS DORSI	0	8
		LONGISSIMUS THORACICAE	1	22
		SERRATUS VENTRALIS THORACIS.	0	0
**LEFT ELBOW**	9	DELTOIDEUS	0	5
		BRACHIORADIALIS	0	0
		TRICEPS BRACHII	1	0
		BRACHIALIS	0	0
		BICEPS BRACHII	0	0
		OMOTRANSVERSARIUS	0	0
**LEFT CARPUS**	0	EXTENSOR CARPI RADIALIS	1	0
		EXTENSOR DIGITORUM COMMUNIS	0	0
		EXTENSOR CARPI ULNARIS	0	0
		FLEXOR CARPI ULNARIS	0	0
**RIGHT SHOULDE R**	0	MASSETER	0	**0**
		BRACHIOCEPHALICUS	0	**1**
		STERNOCEPHALICUS	0	**0**
		TRAPEZIUS PARS CERVICALIS	0	**0**
		PECTORALES SUPERFICIALIS	0	1
		PECTORALIS PROFUNDUS	0	0
		SUPRASPINATUS	0	0
		INFRASPINATUS	0	0
		TRAPEZIUS PARS THORACICA	0	0
		LATISSIMUS DORSI	0	8
		LONGISSIMUS THORACICAE	0	24
		SERRATUS VENTRALIS THORACIS.	0	0
**RIGHT ELBOW**	6	DELTOIDEUS	0	2
		BRACHIORADIALIS	0	0
		TRICEPS BRACHII	1	1
		BRACHIALIS	0	0
		BICEPS BRACHII	0	0
		OMOTRANSVERSARIUS	0	0
**RIGHT**	0	EXTENSOR CARPI	0	0

**CARPUS**		RADIALIS		
		EXTENSOR DIGITORUM COMMUNIS	0	0
		EXTENSOR CARPI ULNARIS	0	0
		FLEXOR CARPI ULNARIS	0	0
**LEFT HIP**	12	LONGISSIMUS LUMBORUM	5	12
		GLUTEUS SUPERFICIALIS	0	0
		GLUTEUS MEDIUS	3	2
		PECTINEUS	1	3
		ILIOPSOAS	0	0
**LEFT STIFLE**	14	BICEPS FEMORIS	0	2
		SARTORIUS	0	3
		TENSOR FASCIAE LATAE	0	0
		QUADRICEPS FEMORIS	7	9
		GRACILIS	0	0
		SEMITENDINOSUS	0	0
		SEMIMEMBRANOSUS	0	0
**LEFT TARSUS**	1	PERONEUS LONGUS	0	0
		PERONEUS BREVIS	0	0
		FLEXOR DIGITORUM SUPERFICIALIS	0	0
		FLEXORES DIGITORUM PROFUNDI	0	0
		GASTROCNEMIUS	0	0
		EXTENSOR DIGITORUM LONGUS	0	0
		TIBIALIS CRANIALIS	0	0
		FIBULARIS	0	0
**RIGHT HIP**	12	LONGISSIMUS LUMBORUM	2	13
		GLUTEUS SUPERFICIALIS	0	0
		GLUTEUS MEDIUS	0	1
		PECTINEUS	2	1
		ILIOPSOAS	0	1
**RIGHT STIFLE**	15	BICEPS FEMORIS	1	1
		SARTORIUS	0	3
		TENSOR FASCIAE LATAE	0	0
		QUADRICEPS FEMORIS	11	8
		GRACILIS	0	1
		SEMITENDINOSUS	0	2

		SEMIMEMBRANOSUS	0	0
**RIGHT TARSUS**	0	PERONEUS LONGUS	0	0
		PERONEUS BREVIS	0	0
		FLEXOR DIGITORUM SUPERFICIALIS	0	0
		FLEXORES DIGITORUM PROFUNDI	0	0
		GASTROCNEMIUS	0	1
		EXTENSOR DIGITORUM LONGUS	0	0
		TIBIALIS CRANIALIS	0	1
		FIBULARIS	0	0
